# Modeling biomarker kinetics of Aβ levels in serum following blast

**DOI:** 10.3389/fneur.2025.1548589

**Published:** 2025-04-04

**Authors:** Carly Norris, Harsha T. Garimella, Walter Carr, Angela M. Boutté, Raj K. Gupta, Andrzej J. Przekwas

**Affiliations:** ^1^Biomedical, Energy, and Materials Division, CFD Research Corporation, Huntsville, AL, United States; ^2^Blast-Induced Neurotrauma Branch, Center for Military Psychiatry and Neuroscience, Walter Reed Army Institute of Research (WRAIR), Silver Spring, MD, United States; ^3^US Army Medical Research and Development Command, DoD Blast Injury Research Coordinating Office (BIRCO), Fort Detrick, MD, United States

**Keywords:** Aβ42, diagnostics, blast, brain, biomarker, serum, modeling

## Abstract

Elucidating the unique neuropathological response to blast exposure remains a barrier towards the development of diagnostic approaches for those with blast-induced traumatic brain injury (bTBI). Quantification of biomarker concentrations in the blood post-injury is typically used to inform brain injury severity. However, injury progression and associated changes in biomarker concentrations are sensitive to parameters such as the blast overpressure (BOP) magnitude and frequency of blast exposure. Through this work, a blast-dose biomarker kinetics (BxK) platform was developed and validated for Aβ42 as a promising predictor of injury post-blast. Blast-dose responses accounting for BOP magnitude and frequency were integrated into a mathematical model accounting for whole-body Aβ peptide kinetics. Validation of the developed model was performed through comparison with acute monomer levels in the blood serum of 15 service members exposed to repeated low-level blast while undergoing three-day weapons training. Amyloid precursor protein (APP) synthesis was assumed to be proportional to blast magnitude and additive effects within a window of recovery were applied to account for cumulative exposure. Aβ42 concentrations in the blood serum were predicted within 6.5 ± 5.2% on average, demonstrating model feasibility and biomarker sensitivity to blast. Outcomes discuss how modulation of patient-specific factors (age, weight, genetic factors, years of exposure, sleep) and pathophysiological factors (BBB permeability, amyloidogenic pathology, neuroinflammation) can reveal potential sources of variability in experimental data and be incorporated into the blast-dose BxK platform in future iterations. Advancements in model complexity accounting for sex-specific factors, weapon system, stress levels, risk of symptom onset, and pharmacological treatment strategies are anticipated to improve model calibration. Utilization of this blast-dose BxK model to identify drivers of pathophysiological mechanisms and predict chronic outcomes has the potential to transform bTBI diagnostic, prognostic, and therapeutic strategies.

## 1 Introduction

Blast-induced traumatic brain injuries (bTBI) and their associated subclinical effects are highly variable and a clear diagnostic platform has yet to be developed, leaving affected military personnel susceptible to increased neurocognitive, behavioral, and functional deficits (1–5). A Generalized Blast Exposure Value (GBEV) was developed by Modica et al. (6) to establish a blast exposure threshold where the probability of developing blast-related symptomology in military Veterans was found to be dependent on the number of overpressure exposures, weapon type, timeframe between first and last exposure, and repetitive daily frequency. Blood proteomics and metabolomics were then correlated with GBEV, which was intended to facilitate the development of interventions for service members (7). Unfortunately, GBEV does not account for blast overpressure (BOP) magnitude or the time between exposures, which are critical metrics towards understanding how low-level vs. high-level and single vs. repeated blast exposures contribute to chronic symptomology, particularly during military training exercises (4, 8–10).

Increased susceptibility to prolonged recovery following repeated impact injury was proposed to be a function of the window of vulnerability (11). The window of vulnerability becomes breached when the new injury occurs prior to the resolution of the pathophysiological recovery from the first injury. Development of an injury model parameterized to account for the recovery phase and associated window of vulnerability following blast could improve predictive pathophysiology.

The National Institute of Neurological Disorders and Stroke (NINDS) recently proposed multidimensional classifications of traumatic brain injury (TBI) severity accounting for clinical presentation, protein biomarkers, anatomical features, and biopsychosocial-ecological factors (12, 13). This new clinical, biomarkers, and imaging with modifiers (CBI-M) framework was developed to enable more effective TBI management (14). Among the CBI-M, clinical predictors of TBI pathophysiology can currently be estimated through quantification of blood biomarker concentrations (15–17). Although the mechanism of injury is different from impact TBI, blood biomarker concentrations have been shown to significantly change following blast, indicating that this method may have strong prognostic and diagnostic capabilities for bTBI applications (18, 19). Determination of the effects of repeated blast exposure on biomarker kinetics, particularly within the window of vulnerability, is a necessary step towards identifying bTBI thresholds and establishing protocols to prevent prolonged cognitive deficits, even following exposure to low-level blasts during training exercises.

Evidence of amyloidogenic processes have been observed in patients with TBI shortly after injury via quantification of amyloid-β peptide 40 (Aβ40) and amyloid-β peptide 42 (Aβ42) monomers in the peripheral blood (20, 21). Aβ peptides are produced from cleavage of amyloid precursor protein (APP) found in high concentrations within the neuronal cytoplasm and at synaptic terminals. Following exposure to low-level blast, Aβ40 and Aβ42 were both elevated, associated with symptomology at acute and chronic timepoints. However, Aβ42 has been shown to have greater sensitivity compared to Aβ40 as a biomarker for bTBI (21–25). Therefore, the purpose of this study was to develop a model capable of predicting brain biomarker kinetics of Aβ42 following blast exposure.

Aβ42 release kinetics were previously simulated for single versus repeated blast exposure cases corresponding to degree of synaptic damage where results showed approximate windows for biomarker collection between 2 and 5 days (26). Prediction and validation of Aβ42 biomarker dynamics based on previous experimental and computational findings in this proof-of-concept study aims to inform how blast mechanics influence pathophysiology where we intend to expand upon future models to improve diagnostic and prognostic capabilities.

## 2 Materials and methods

**Figure 1:**
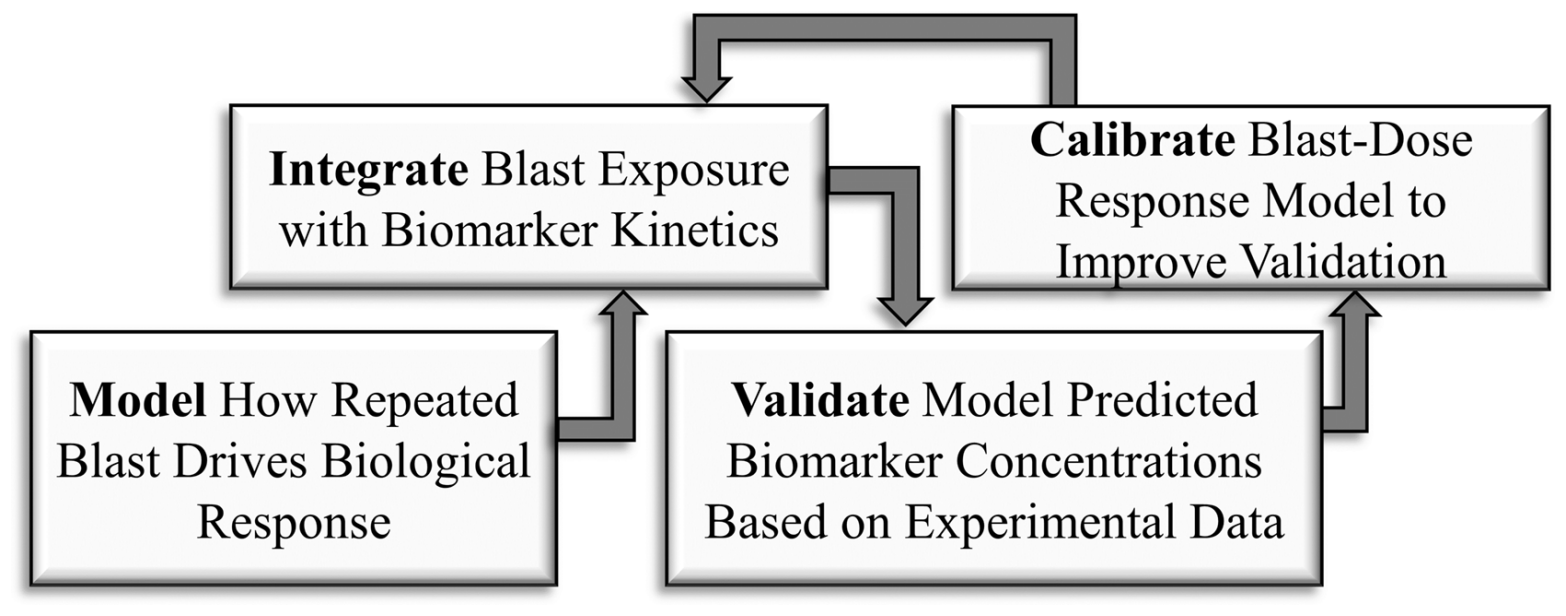
Schematic of model development approach: (1) model, (2) integration, (3) validation, and (4) calibration.

Blast-dose biomarker kinetics (BxK) model development can be divided into four main segments (Figure 1). A system of first-order ordinary differential equations (ODEs) was parameterized to describe magnitudes and durations of cellular mechanical damage (milliseconds to seconds) and associated phasic biological responses (minutes to hours). Mechanical damage was conceptualized as the phase in which external forces alter baseline solid or fluid mechanics, sometimes referred to as the primary injury. Phasic biological responses correspond to biochemical processes resulting from the initial injury, which can also be defined as secondary injury cascades (27). Parameter estimation and model formulation was adapted from previous models describing synaptic mechanobiology and biomarker responses (26). Blast-dose responses accounting for BOP magnitude and frequency were then integrated into a mathematical model accounting for whole-body Aβ peptide kinetics. Next, validation of the model was performed through comparison of predicted and measured serum Aβ42 concentrations following blast exposure in 15 soldiers. Lastly, the developed BxK model discusses avenues for model calibration to improve predictive capabilities, establishing a framework that could be expanded for other biomarkers.

### 2.1 Modeling blast-dose and biological response

During blast exposure, complex cellular environments are subjected to high strain rates. Animal, surrogate, and computational models typically approximate tissue biomechanics as a function of BOP magnitude (28–33). Thus, mechanical energy deposition and subsequent mechanical damage ( $R_{M}$ ) were assumed to be diffuse throughout the whole brain and proportional to the magnitude of the BOP. The associated recovery rate was represented as a linear kinetic term (Equation 1):


(1) $$\frac{dR_{M}}{dt}=-\frac{\alpha }{t_{d}}\left(R_{M}-\lambda \right)$$

**Figure 2:**
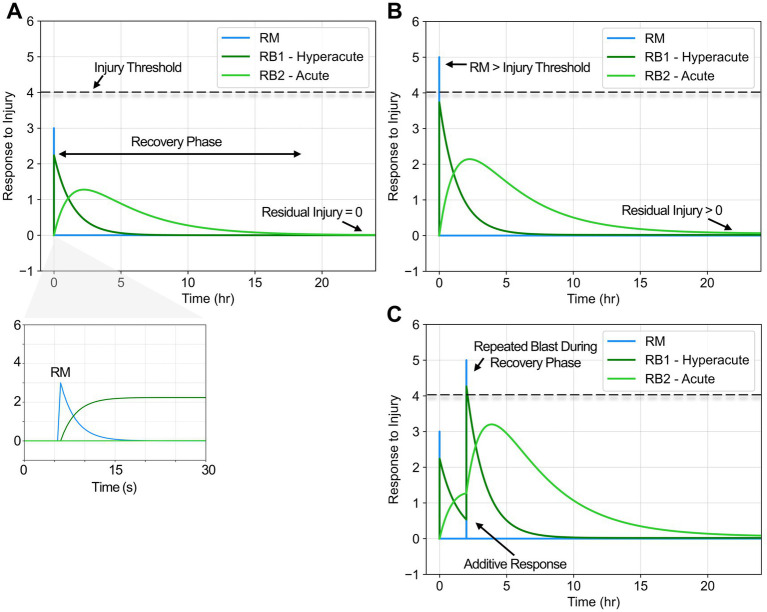
Modeling mechanical damage ( $R_{M}$ ) and multi-phase biological responses ( $R_{B1}$ and $R_{B2}$ ) to blast exposure. **(A)** Biological responses to a blast magnitude below the injury threshold normalize to zero, representing full recovery. Time to peak $R_{M}$ was ~500 ms. **(B)** Biological responses to a blast magnitude above the injury threshold no longer normalize to zero, representing residual injury proportional to BOP magnitude. Figure annotations adapted from Blennow et al. (11). **(C)** If a repeated blast occurs within the recovery phase, the response was assumed to be additive.

$R_{M}$ is the mechanical damage to brain microstructures, *α* is the damage decay rate, $t_{d}$ is the characteristic time for the impulse duration of mechanical damage, and *λ* represents the residual damage. The model assigns an injury threshold, which drives $R_{M}$ . In this study, the non-dimensional injury threshold was set to 4, proportional to overpressures of 4 psi where pressures exceeding this threshold were linked to low-level blast pathophysiology (4, 34, 35). However, this threshold can be easily adjusted within the framework to be proportional to alternative input parameters (i.e., positive phase impulse, cumulative impulse, intensity, etc.). The time to peak mechanical damage was assumed to be on the order of 500 ms. While dynamic loading due to blast overpressures or impact typically occurs on the order of 1–50 ms, shear waves may last hundreds of milliseconds post blast (36, 37). Below the assigned injury threshold, the residual mechanical damage (*λ*) was assumed to approach zero, simulating full recovery (Figure 2A). On the other hand, above the injury threshold, residual damage approaches a constant proportional to the blast magnitude (λ ~ BOP), simulating chronic damage (Figure 2B).

Molecular responses to tissue damage within the brain can be parameterized through multiple phases. The initial or hyperacute response is typically defined by molecular changes occurring within the first seconds to hours following injury. Subsequent phases include acute (<24 h), subacute (1 day–3 weeks), and chronic (>3 weeks), although exact time frames are widely disputed (38, 39). In this work, hyperacute $\left(R_{B1}\right)$ and acute $(R_{B2}$ ) phase biological responses were defined as functions of the mechanical damage, $R_{M}$ , imposed by the blast according to Equations 2, 3):


(2) $$\frac{dR_{B1}}{dt}=k_{\mathrm{in}}R_{M}-k_{\mathrm{out}1}R_{B1}\left(R_{M}+R_{M0}\right)$$

(3) $$\frac{dR_{B2}}{dt}=k_{\mathrm{out}1}R_{B1}-k_{\mathrm{out}2}R_{B2}$$

where $k_{\mathrm{in}},k_{\mathrm{out}1}$ , and $k_{\mathrm{out}2}$ are constants controlling the rate of change of neurobiological responses and $R_{M0}$ is the basal mechanical repair (assumed $R_{M0}$ = 1). Representative hyperacute and acute responses to single and repeated blast exposure are shown in Figure 2 to demonstrate how mechanical damage drives the cascade of biological responses. If the repeated blast occurs within the recovery phase, the mechanical damage and biological responses were assumed to be additive (Figure 2C), accumulating any residual damage at the onset of a repeated blast exposure.

### 2.2 Integration with BxK model

**Figure 3:**
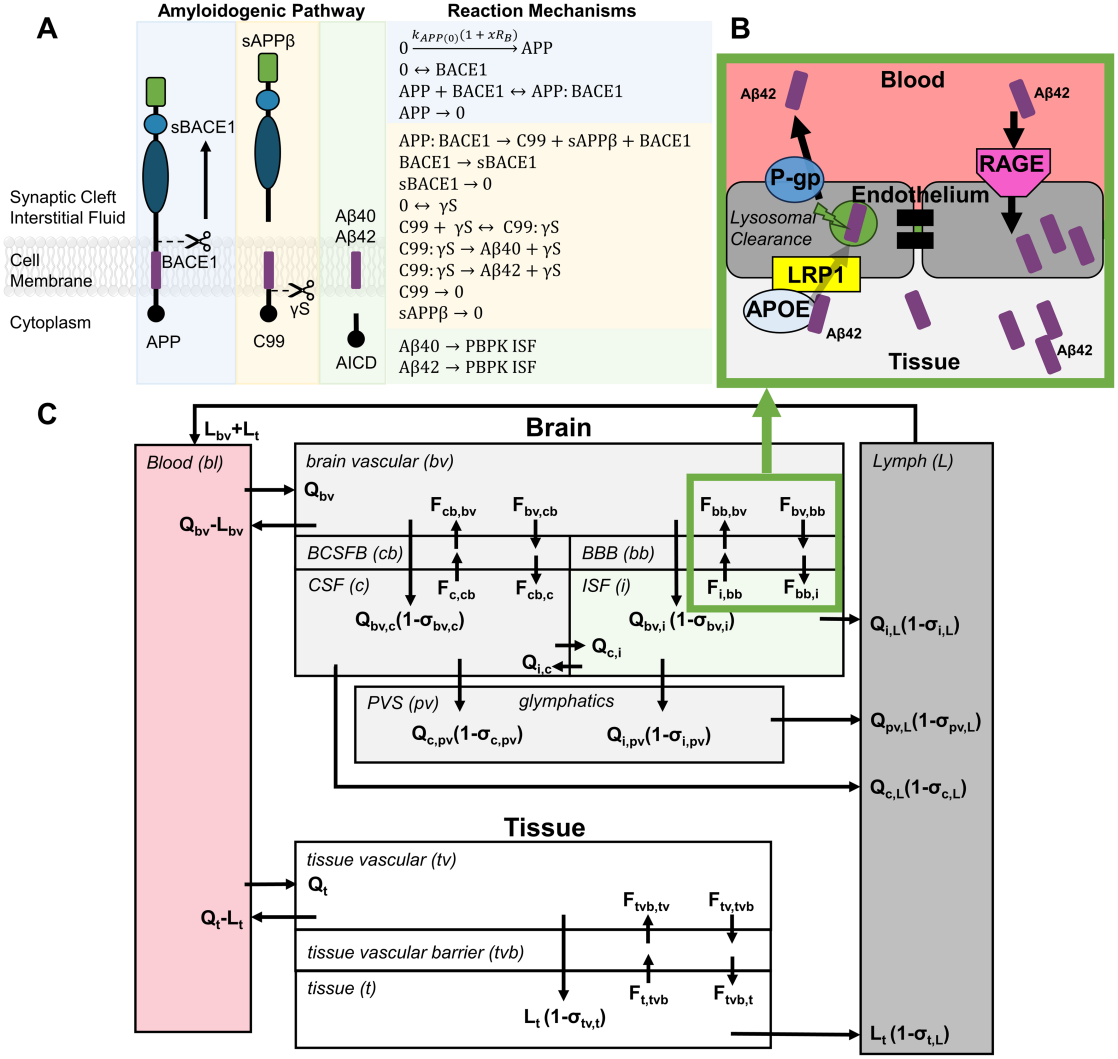
Integration of blast-dose response model with Aβ biomarker kinetics. **(A)** The blast-dose biological responses $\left(R_{B}\right)$ drive APP synthesis in the amyloidogenic pathway, affecting Aβ monomer concentrations in the interstitial fluid (ISF). **(B)** Aβ peptide flux between tissue and blood was defined to include receptor-mediator endocytosis and bi-directional transport. **(C)** Biomarker concentrations in the serum were predicted using a PBPK model adapted from Bloomingdale et al. (44), incorporating exchange between brain, whole body tissue, lymph, and blood compartments.

Amyloid precursor protein (APP) is typically metabolized through the non-amyloidogenic pathway at the plasma membrane where soluble p3 peptides are effectively cleared from the body (40). However, when APP is cleaved through the amyloidogenic pathway, toxic peptides Aβ40 and Aβ42 aggregate from monomers into dimers, trimers, oligomers, and protofibrils and fibrils develop into Aβ plaques (41). Aβ monomers are formed through the following steps in the amyloidogenic pathway (Figure 3A): (1) APP is cleaved by beta-site APP cleaving enzyme 1 (BACE1) at the cell membrane, generating sAPPβ and C99. BACE1 may then be cleaved to form a soluble fragment (sBACE1). (2) C99 is enzymatically broken down by *γ*-secretase (γS) into Aβ peptides and APP intracellular domain (AICD). Aβ40 and Aβ42 are highly lipophilic and have a propensity for aggregation. They are distinguished by their peptide length of either 40 or 42 amino acids depending on where they were cleaved and are released into the interstitial fluid (ISF).

Change in APP level following exposure to overpressures between 5 and 12 psi was positively correlated to the overpressure magnitude, indicating that blast disrupts APP metabolism (42, 43). Provided that both Aβ40 and Aβ42 were shown to be elevated in the peripheral blood following exposure to low-level blast (21–24), we assumed that under blast conditions, APP was more likely to be cleaved through the amyloidogenic pathway, resulting in increased production of Aβ peptides at the neuronal cell membrane and subsequent release into the ISF surrounding the synapse. To account for this assumption, the rate of APP synthesis ( $k_{\mathrm{APP}}$ ) was increased proportional to the biological response phase post-blast (Equation 4):


(4) $$k_{\mathrm{APP}}=k_{\mathrm{APP}\left(0\right)}\left(1+x\;R_{B}\right)$$

where *x* was a constant determined during model calibration.

Given the complexity of factors influencing biomarker concentrations post-blast, a BxK model was developed to best predict Aβ concentrations in the blood serum. A physiologically based pharmacokinetic (PBPK) model, originally developed in Bloomingdale et al. (44) to model antibodies targeting the central nervous system, was adapted in this study. PBPK models have advantages over typical compartment models where systems of equations account for detailed physiological processes, such as Aβ generation and transport mechanisms. Aβ transport was simulated by adapting the typical vascular-endothelial-interstitial barrier model described by Chang et al. (45) where IgG antibodies in the vasculature were modeled to bind to FcRn, enter the vascular endothelial cells via fluid-phase endocytosis, and be taken up by endo-lysosomes for partial degradation and release into both the vasculature and interstitium. In this work, Aβ peptide transport was adjusted to account for receptor-mediated endocytosis of Aβ and bi-directional fluid transport across barriers (Figure 3B) (46). Aβ transport is facilitated by various influx and efflux transporters. Low-density lipoprotein receptor-related protein 1 (LRP1) and P-glycoprotein (P-gP) transporters control the Aβ efflux from interstitium to vasculature, while the receptor for advanced glycation end-products (RAGE) transporter controls the Aβ influx from vascular to interstitial space (46–48). Additionally, compartment models were further expanded to account for exchange between the brain, perivascular space, glymphatics, peripheral tissues, plasma, and lymph (Figure 3C). Reaction mechanisms were formulated as mass balance equations between compartments where the tissue flow rates (Q), lymphatic flow rates (L) and exchange flux rates (F) describe mass transport. Amyloidogenic reaction mechanisms (Figure 3A), PBPK reaction mechanisms (Supplementary Table S1), parameter constant assumptions, and initial conditions (Supplementary Table S2) were used to generate and solve complex systems of ODEs using the CoBi (CFDRC Computational Biology tools) ODE-Gen module. Model simulation was then performed using CoBi software (49).

### 2.3 Validation

**Figure 4:**
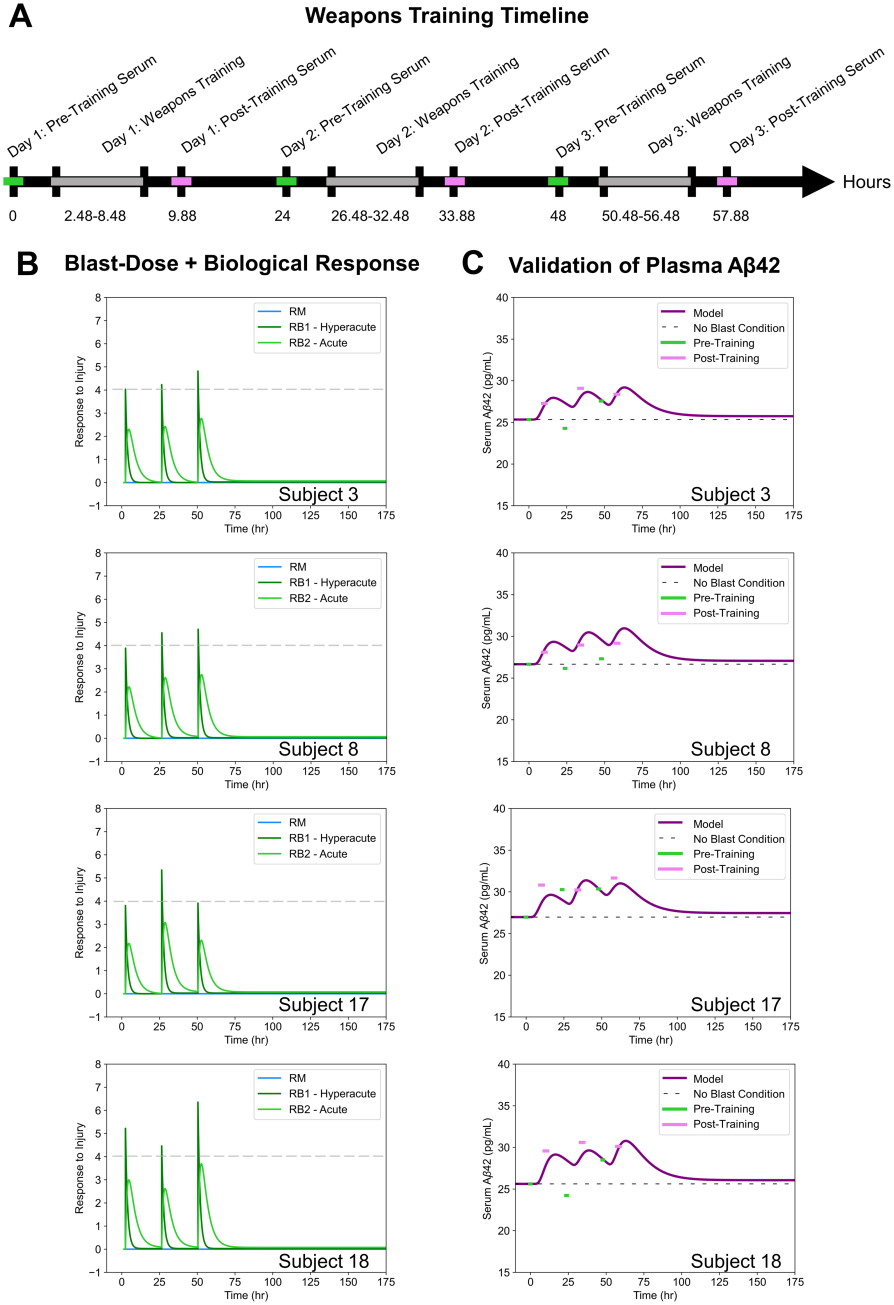
**(A)** Weapons training timeline based on assumptions from Thangavelu et al. (24). **(B)** Blast-dose + biological response curves for four subject-specific cases. **(C)** Blast-Dose BxK model serum Aβ42 concentrations compared to experimental serum data for four subjects.

Biomarker concentrations in the blood serum of male Soldiers (n = 15) were collected over the course of a three-day 0.50-caliber sniper rifle training in a study by Thangavelu et al. (24). The investigators have adhered to the policies for protection of human subjects as prescribed in AR 70–25. Serum was collected 2.1–3.16 h prior to weapons training (2.48 h on average) where it was assumed that weapons training lasted a total of 6 h each day. Post-training serum was collected 0.48–2.90 h after training (1.40 h on average). A weapons training timeline was generated based on this information (Figure 4A).

Soldiers were exposed to 4–50 shots fired per day while instrumented with sensors in a location on their body that approximated individualized incident overpressure exposures throughout training. Average BOP magnitude per day, as provided in the experimental dataset, was input into our blast-dose model to recreate the patient-specific $R_{M}$ and $R_{B}$ (Figure 4B). The effect of the hyperacute biological response ( $R_{B1}$ ) on blood serum Aβ42 concentrations was analyzed throughout this study.

Predicted serum biomarker concentrations were normalized to the subject-specific pre-training concentration on day one of weapons training. Relative percent error was calculated between the time-course profile of model Aβ42 concentrations in the serum and the reported subject-specific Aβ42 concentrations before and after each training day (six data points per subject). A two-tailed Wilcoxon paired t-test was used to statistically compare accuracy of model predictions at the beginning of each training day versus the end of each training day where *p* < 0.05 was considered significant. The effect of age and duration of service on model accuracy was also assessed using linear regression analysis.

## 3 Results

### 3.1 Model performance

The relative percent error of the model at each time point was calculated and reported for all patients in Table 1 as a measure of model accuracy. Since all model predictions were normalized to the baseline serum levels on day one, the relative percent error at *Day 1: Pre-Training* was zero in all cases and was not included in the analysis. Representative comparisons from model outputs from four blast scenarios are provided for discussion (Figure 4C). Outputs from all 15 subject-specific scenarios are shown in Supplementary Figure S1. Qualitatively, it is evident that post-training experimental data points (pink) most closely align with the predicted model compared to the pre-training experimental data points (green).

**Table 1:** Relative percent errors of the predicted serum Aβ42 concentrations in the blast-dose BxK model compared to experimental data at each time point during the three-day weapons training. Person-specific information from Thangavelu et al. (24).

Person-specific information			Serum Aβ42 relative % error				
Subject ID	Age	Duration of service (years)	Day 1: post-training	Day 2: pre-training	Day 2: post-training	Day 3: pre-training	Day 3: post-training
1	33	11	0.9	20.2	1.3	13.4	7.0
3	34	10	−1.7	12.3	−4.2	0.2	−0.4
4	46	19	−11.7	11.8	−5.0	1.7	−6.6
6	50	22	2.0	20.0	1.9	9.7	8.3
7	47	26	−5.2	2.5	7.4	6.7	−3.5
8	43	18	0.2	9.5	1.7	8.0	3.1
9	35	8	−3.2	5.8	−4.2	8.6	−1.0
11	43	9	−6.3	15.6	−0.8	11.9	0.5
12	37	12	−7.2	−2.3	2.5	7.1	−3.4
14	52	10	−6.2	17.3	−0.4	10.3	7.9
16	46	19	−16.2	14.4	−6.0	−11.8	−1.2
17	45	15	−7.7	−4.3	−0.5	−0.2	−4.0
18	36	8	−6.8	17.1	−5.6	0.0	−1.8
19	42	12	3.1	17.3	4.3	13.8	8.6
20	46	19	−12.1	3.1	−7.4	−6.3	−2.9

**Figure 5:**
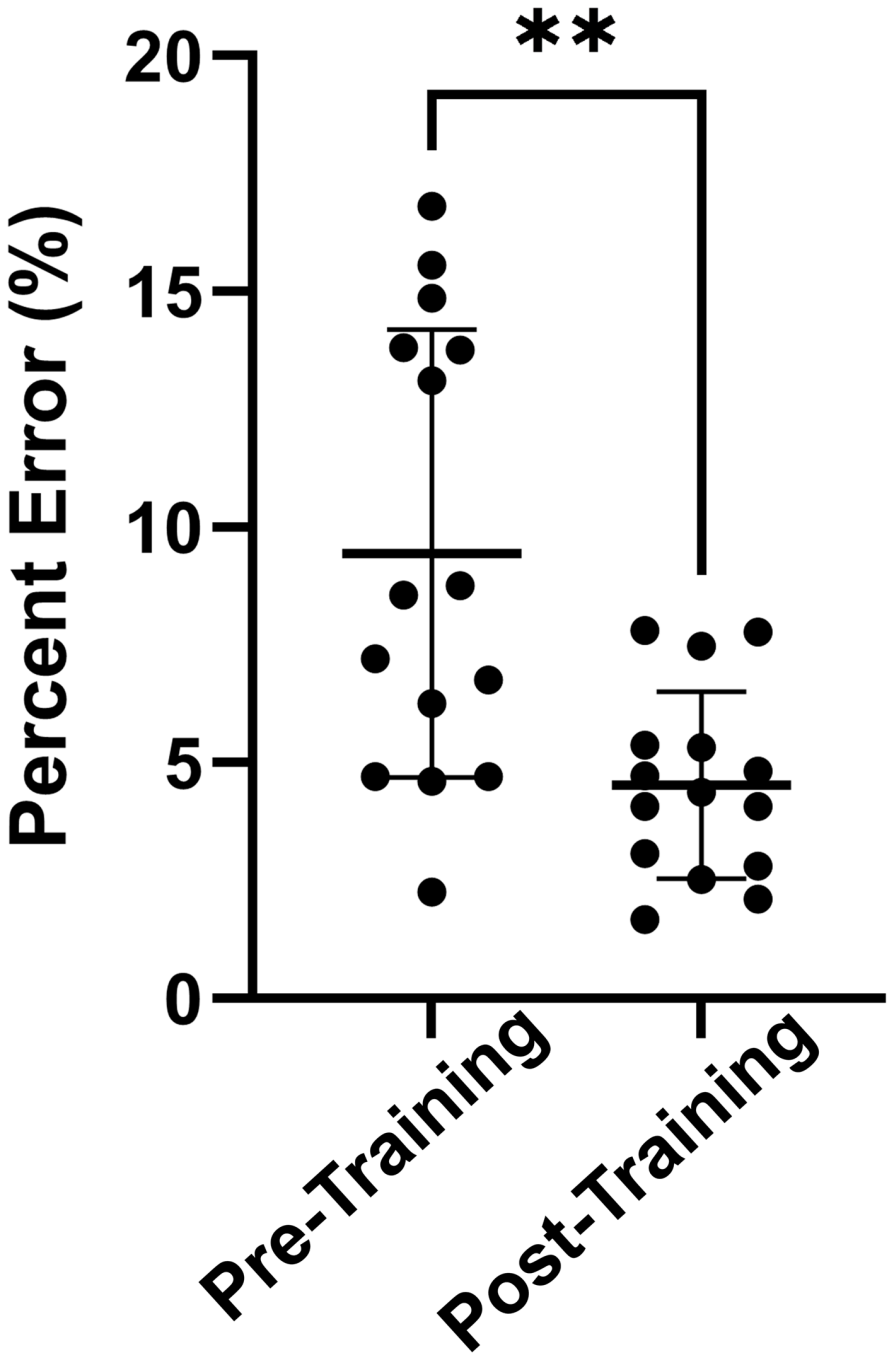
Absolute percent error of average pre-training and post-training model predictions for each person. Pre-training model predictions had significantly greater percent error compared to experimental data than post-training model predictions (**p < 0.01). Statistics were calculated using a two-tailed Wilcoxon paired t-test. Results are displayed as Mean ± SD.

The predicted change in serum Aβ42 concentrations over time resulted in a mean error of 6.5 ± 5.2%, demonstrating high accuracy of the blast-dose BxK model. Post-training predictions over the three-day training were significantly better than pre-training predictions (*p* < 0.01) (Figure 5). Further, 80% of pre-training data points were over-approximated. Regression coefficients comparing subject age or duration of service and degree of model pre-training predictions were less than 0.1, indicating that alternative factors are likely driving variability in subject pre-training Aβ42 levels.

### 3.2 Calibration

Increased APP synthesis proportional to the blast magnitude resulted in an increase in Aβ monomer concentrations in the serum. Calibration of the model established *x* = 1.5 in Equation 4, which was applied for all subjects in this study. However, future iterations of the blast-dose BxK model may be optimized to determine a person-specific *x* based on factors such as age, duration of service, sex, etc. A more in-depth discussion of future calibration and model capabilities is provided in the Discussion section.

## 4 Discussion

The blast-dose–response BxK model predicted serum Aβ42 concentrations as a function of BOP magnitude within 6.5% error on average. A hyperacute biological response was applied to APP synthesis and time-course profiles within the acute phase of injury were validated based on experimental data. Over-approximation of pre-training concentrations contributed to the greatest percent error within the model. Experimental data showed that biomarker concentrations returned to baseline by day two or even decreased (24), which is consistent with preclinical results at 24 h following blast (43). De Gasperi et al. found that despite increased APP production following blast, Aβ42 was still decreased at 24 h (43). The physiological explanation for this decrease remains unclear. However, factors affecting model variability could be due to limitations in the model assumptions. In particular, the assumed rate of neurobiological response or basal repair may be sub-optimal. Acute responses to blast exposure leading to amyloid suppression should be investigated/incorporated into the model.

### 4.1 Limitations

#### 4.1.1 APP synthesis assumptions

The BxK model simulated response to blast based on the assumption that blast drives APP synthesis within brain tissue. This led to increased Aβ42 concentrations due to enzymatic cleavage. However, the source of increased Aβ peptides in serum after blast remains unclear. Increased levels of Aβ42 may also occur due to (1) increased cleavage/clearance by platelet activation in the whole blood leading to an increase in Aβ42 in the serum following blast, and/or (2) increased enzymatic cleavage/clearance through the blood brain barrier (BBB) from blast-related damage (sourced either from the blood or from the brain tissue) (50–54). Mechanistic studies can be employed using these models to test these hypotheses.

The rate of APP synthesis in the peripheral tissues was assumed to be 10% of initial APP synthesis in the brain ( $k_{\mathrm{APP}\left(0\right)}$ defined in Eq. 4). This was based on the assumption that >90% of APP concentrations brain are platelet-derived (54). However, model APP synthesis rates and concentrations of brain tissue-derived, platelet-derived, and peripheral tissue-derived APP (from the pancreas, kidney, spleen, heart, liver, lung, intestines, skin, and salivary and thyroid glands) should be calibrated in greater detail prior to making any mechanistic conclusions of how blast may be contributing to changes in Aβ concentrations (47, 54, 55).

#### 4.1.2 Validation

The number of subjects used to validate this model (*n* = 15) was sufficient to demonstrate model feasibility and application. Nevertheless, validation of this model using larger subject populations is necessary to make any mechanistic conclusions of how blast may be contributing to changes in Aβ concentrations.

Using the model framework, modulation of fluid exchange between the cerebrospinal fluid (CSF), ISF, BBB, and lymphatic systems to improve model fit within the first 24 h post-injury could be one way to illuminate the potential factors affecting Aβ42 kinetics. Additional factors, such as stress levels or sleep, could have also influenced clearance of these biomarkers. Therefore, improved model predictions of inter-day recovery likely require incorporation of more person-specific factors. The current blast-dose BxK model predictions demonstrate feasibility; however, the following person-specific and pathophysiological factors can be readily incorporated to improve predictive capabilities.

### 4.2 Person-specific factors

#### 4.2.1 Age

Aβ42 concentrations are known to vary with age. Healthy subjects aged 35–65 had significantly higher Aβ42 plasma levels than subjects less than 35 (56). This gradual increase in baseline concentrations with age was incorporated into our model, however, we were most interested in predicting the relative acute change in concentration of monomers within this study. As the effect of bTBI on the Aβ42 production with age remains unknown, future applications of this model may account for chronic changes in monomer concentration, amyloid suppression processes, and/or age-specific amyloidogenic pathology contributing to dimerization, oligomerization, and eventually plaque formation.

#### 4.2.2 Body weight

Cardiac output, hematocrit, whole blood volume, serum volume, clearance, bioavailability, and flow rates are typically variables either calculated from body weight or normalized to body weight, which should be tailored to the individual. In the validation dataset, the body weights were unreported, which could contribute to variability in the predicted model. Incorporation of experimental datasets reporting body weight may allow for improved predictive modeling.

#### 4.2.3 Genetic risks

Humans expressing the apolipoprotein E4 (APOE4) genotype are known to have an increased genetic risk of developing Aβ pathology over time (57, 58). Presentation of APOE4 can also increase neuroinflammation and reduce Aβ clearance through the BBB (57). The current study focuses on predicting the change in Aβ42 monomer concentrations corresponding to blast magnitude and frequency. However, antibody complexes and macrophage-based clearance for larger plaques are also incorporated into the model framework (59). Therefore, if the patient genotype is known, the assumption that APOE4 carriers have decreased macrophage-based clearance compared to noncarriers may be applied to the blast-dose BxK model to examine the potential effects following blast injury.

#### 4.2.4 Years of exposure

Aβ42 levels were previously correlated with years of service (23). On the other hand, experimental data from Thangavelu et al. (24) did not show any association with duration of service. Blast exposure history assessment instruments, such as GBEV, may provide a more complete measure accounting for potential underlying pathology from chronic exposure (6). For instance, military personnel receive a score based on responses to questions asking about exposure in terms of years over lifetime, months per year, days per month, explosions per day, and frequency of multi-day exposures. One way that GBEV could be incorporated into the blast-dose BxK model could be as a predictor of the residual damage, proportional to *λ* in Equation 1. Calibration of the model using GBEV, or a comparable measure of blast exposure history, would better inform subject-specific injury thresholds and recovery periods for Aβ42 along with other biomarkers if any are shown to be sensitive to blast exposure.

#### 4.2.5 Sleep

Aβ deposition and glymphatic clearance can be significantly influenced by sleep cycles. Sleep duration, quality, and intraday variability are common sleep measures associated with Aβ pathology (60, 61). Aβ accumulation occurs when there is decreased clearance of Aβ through the glymphatic system (62, 63). The deviation between model prediction of Aβ42 and measured levels on the morning following blast may be explained through further investigation of amyloid levels in the brain and serum associated with sleep–wake cycles, diurnal patterns, or rest phases. Therefore, incorporation of the glymphatic system and altered glymphatic flux based on patient-specific sleep patterns should be incorporated in future model iterations.

### 4.3 Pathophysiological factors

#### 4.3.1 BBB permeability

Neurons, astrocytes, and the BBB are tightly coupled such that when blast loading and associated high strain rates are experienced throughout the brain, damage to the neuronal and astrocytic membranes contributes to the release of intracellular proteins into the interstitial space. Increased permeability of the BBB occurs following blast where the temporal changes in permeability were also found to be dependent on BOP magnitude (52, 64, 65). Large proteins, typically not found in high concentrations in the blood, are able to diffuse into the bloodstream through enlarged spaces at the tight junctions between endothelial cells of the BBB, caused by untethering of adhesion molecules under loading (16, 66). The sensitivity of Aβ42 in the serum, particularly at low blast exposures, may be related to its small size. Therefore, the concentration of larger neuronal- or astrocyte-specific proteins detected in the blood can indicate the extent of diffuse cellular damage. Current blast-dose BxK model framework can account for BBB permeability between ISF and brain vascular compartments by parameterizing flux across the BBB as a function of blast-dose.

#### 4.3.2 Amyloidogenic pathology

The amyloidogenic pathway is comprised of APP, BACE1, sBACE1, γS, sAPPβ, C99, AICD, and Aβ peptides (Figure 3). The assumption that blast drives an increase in APP synthesis was effective at directly predicting Aβ42 concentrations in the serum. However, there is a need for more experimental data to determine whether BACE1 or γS activity increases with blast proportional to APP. Parametric simulations predicting the effect of changes in these pathway components on serum Aβ concentrations may provide valuable insight to potential therapeutic avenues for bTBI.

#### 4.3.3 Neuroinflammation

The purpose of this study was to validate monomer concentrations of Aβ42 in the serum, however, the blast-dose BxK model directly integrates with additional functions accounting for the Aβ aggregation cascade, antibody binding to Aβ targets, generation of antibody:Aβ complexes, activation of microglia and perivascular macrophages, and their effects on clearance. Simulations predicting likelihood of Aβ plaque formation following blast and modulation of clearance mechanisms could enable investigation of blast pathophysiology and its association as a risk factor for neurodegenerative diseases. Furthermore, given that activation of microglia is a common mechanism of secondary injury contributing to neuroinflammation following blast (67, 68), it is likely that calibration of these parameters based on BOP or impulse in future iterations would improve predictions of acute clearance.

### 4.4 Future considerations

#### 4.4.1 Alternative injury predictors

In this study, BOP magnitude was input and assumed to be directly related to the degree of mechanical damage, which was found to reasonably predict Aβ42 levels. However, alternative predictors of injury severity may have improved associations with alterations in biomarker levels over time. In Thangavelu et al. (24), a measure of the subject-specific summed impulse over the three-day training course was found to directly correlate with *Day 3: Post-Training* Aβ42 levels (correlation coefficient = 1, *p* < 0.0001). Therefore, we originally hypothesized that inputting the average impulse x total shots fired over the course of each training day would be a better predictor of Aβ42 levels than BOP magnitude. However, model predictions had a greater percent error based on this measure compared to BOP magnitude. Nevertheless, it is possible that measures of blast intensity, daily summed impulses, durations, rates of change of pressure, etc. could have improved predictive capabilities for Aβ42 and may even be biomarker-specific.

#### 4.4.2 Sex

The experimental data used to validate our model was based on data collected from all males. Thus, model outputs were based on physiological parameters from a 70 kg male. Sex-dependent PBPK models may be implemented to consider the influence of sex hormones on Aβ42 pathology following blast (69). While there remains no evidence to support sex-specific differences in Aβ42 biomarker concentrations following blast, sex-specific differences have been shown to be a factor in neurodegenerative disease progression (70). Incorporation of sex-dependent responses into the blast-dose BxK model using the PBPK framework would improve personalized diagnostics and inform potential treatment strategies that may be affected by sex-specific physiology.

#### 4.4.3 Weapon system

Validation of the model was performed for a single weapon system (0.50-caliber sniper rifle). However, numerous weapon systems (explosive breaching, mortar, artillery, machine guns, sniper rifles, shoulder launched munitions, etc.) generate overpressures capable of contributing to bTBI pathology. Each weapon system generates signature overpressures, which can be replicated, accounting for the blast loads on military personnel (71). For instance, CoBi-Blast tools effectively generated the weapon signature for 0.50 caliber sniper rifle training scenarios (72). As a wider range of weapon systems are modeled, incorporation of weapon signature-specific blast-dose profiles could improve model sensitivity and allow for modulation of the effect of weapons systems on injury pathophysiology.

#### 4.4.4 Stress

Military personnel are exposed to a variety of chronic operational stressors (lack of sleep, altitude, nutrition, temperature, social displacement, etc.), which can predispose individuals to elevated stress levels prior to injury (73). Additionally, 42% of service members were found to have abnormal hormone levels following blast exposure where untreated post-traumatic hypopituitarism can be associated with numerous cognitive deficiencies, such as post-traumatic stress disorder, and compound bTBI pathology (74). These pre-stress conditions may significantly influence biomarker sensitivity post-blast. Specifically, elevated cortisol levels relative to Aβ42 can significantly increase fibril formation and propagate amyloidogenic pathology (75). Biomarker interactions with stress-specific hormones may be required in future iterations to account for these molecular dynamics, likely affecting long-term deficits.

#### 4.4.5 Mechanobiology

Changes in blood biomarker concentrations as a result of bTBI stems from diffuse cellular damage across multiple cell types (endothelial, astrocytes, neurons, oligodendrocytes, etc.) and subdomains within each cell (synapse, dendritic spines, axon, BBB, astrocyte end feet, etc.). Future development of this model framework aims to better account for multifarious cellular damage. For example, it was assumed that increased APP synthesis led to increased production of Aβ peptides at the neuronal cell membrane and subsequent release into the ISF surrounding the synapse. However, there are cases where APP synthesis can lead to axonal swelling and release of peptides along the axon in addition to the synapse (76). Onset of axonal swelling may be proportional to BOP magnitude, allowing for accumulation of APP and subsequent release of Aβ42 prior to being released from the synapse. The current model also assumes generation of APP from basal levels, but future models could incorporate levels of cumulative APP deposits along the axon, depending on BOP thresholds and windows of vulnerability.

#### 4.4.6 Additional biomarkers

The generation of multi-phasic biological responses for a given blast scenario in this work supports model development for additional biomarkers. Aβ42 was selected in this study due to its sensitivity to BOP magnitude and impulse, however, additional experimental data was provided in Thangavelu et al. where GFAP, Nf-L, and Aβ40 also demonstrated utility as biomarkers following blast (24). Based on the available experimental data, additional biomarker-specific models may be developed and validated, accounting for pathway-specific generation and mechanobiology-specific release.

#### 4.4.7 Non-invasive biofluid diagnostics

Blood-based biomarker collection can be invasive, expensive, and limited to specialty clinical settings. Recently, it was reported that non-invasive collection of biomarkers for TBI may be feasible via the collection of saliva, specifically salivary microRNA (77). Therefore, adaptation of the developed model to account for a salivary compartment may extend the functionality of this tool to assess sensitivity of non-invasive biomarker responses post-blast and improve mechanistic understanding of alternative non-invasive diagnostic strategies.

#### 4.4.8 Symptomology

The developed model framework may be able to predict associations with reported symptomology. Aβ42 was previously associated with reports of ear ringing and memory problems (23). However, alternative datasets have shown a lack of association of serum biomarker levels with patient symptomology. Self-reported symptomology associated with Aβ42 serum concentrations were recorded in Thangavelu et al., reporting symptoms related to the onset of pain, anxiety and depression, sensory, motor, or cognitive deficits (24). Given larger datasets with stronger associations to serum biomarker concentrations and symptoms, future developments of this blast-dose BxK model may be adapted to predict patient-specific likelihood of symptom onset.

#### 4.4.9 Prognosis

Current model predictions are only validated for repeated low-level blasts up to a three-day time point. Validation and calibration of the model based on larger datasets encompassing longer time frames is necessary to achieve full model utility. We propose that determination of the subject-specific injury risk or optimal time frame for clinical assessment could be possible given the known weapon training scenario. Ideally, this predictive tool may be used to inform safe practices in weapons training scenarios to avoid poor prognostic outcomes.

#### 4.4.10 Brain region

Mechanical energy deposition and subsequent mechanical damage were assumed to be diffuse throughout the whole brain. While this is not a bad assumption, molecular responses occur disproportionally throughout different brain regions following blast. The significant metabolic demand of the hippocampus, cortex, and cerebellum leave these regions susceptible. Further refinement of the developed model accounting for brain region-specific mechanisms could improve chronic predictions and aid in the identification of therapeutic targets within these vulnerable regions.

#### 4.4.11 Non-brain tissues

This BxK model considers all non-brain tissues as a single compartment, which limits resolution accounting for mechanistic effects specific to major organ systems, such as the kidney, liver, or spleen (important for Aβ42 elimination) (55) or lungs (important in polytrauma blast injury models) (78). Expansion of this PBPK model into high-resolution peripheral compartments, based on parameters established in German et al. (79), could support mechanistic analysis of how non-brain tissues contribute to changes in biomarker concentrations following blast exposure.

## 5 Conclusion

The blast-dose BxK model developed in this study demonstrates feasibility in predicting Aβ42 kinetics as a function of BOP. Current capabilities of this model allow for mechanistic investigation into factors driving changes in biomarker levels following blast exposure, which is critical for identification and implementation of safeguards for soldiers and civilians. Further expansion of the current framework is anticipated to improve biomarker predictions by accounting for additional variables (weapon system, person-specific factors, BBB permeability, etc.). Outcomes from this study highlight an avenue towards elucidating bTBI mechanisms, identifying sensitive biomarkers and diagnostics, and developing effective treatment strategies.

## Data Availability

The raw data supporting the conclusions of this article will be made available by the authors, without undue reservation.

## Author’s note

This research was conducted prior to Boutte’s employment at the NIH. The opinions expressed in this article are the author’s own and do not reflect the view of the National Institutes of Health, the Department of Health and Human Services, or the United States government. Material has been reviewed by the Walter Reed Army Institute of Research, and Department of Health and Human Services. There is no objection to its presentation and/or publication. The opinions or assertions contained herein are the private views of the author, and are not to be construed as official, or as reflecting true views of the Department of the Army or the Department of Defense or the Department of Health and Human Services.
