# Supplementary material for: Modeling biomarker kinetics of Aβ levels in serum following blast
Source: Front Neurol. 2025 Apr 4;16:1548589. doi: 10.3389/fneur.2025.1548589 (PMC12006977; doi:10.3389/fneur.2025.1548589)
Supplement: Supplementary file 1 [file Data_Sheet_1.docx]

Supplementary Material

# Supplementary Figures

#
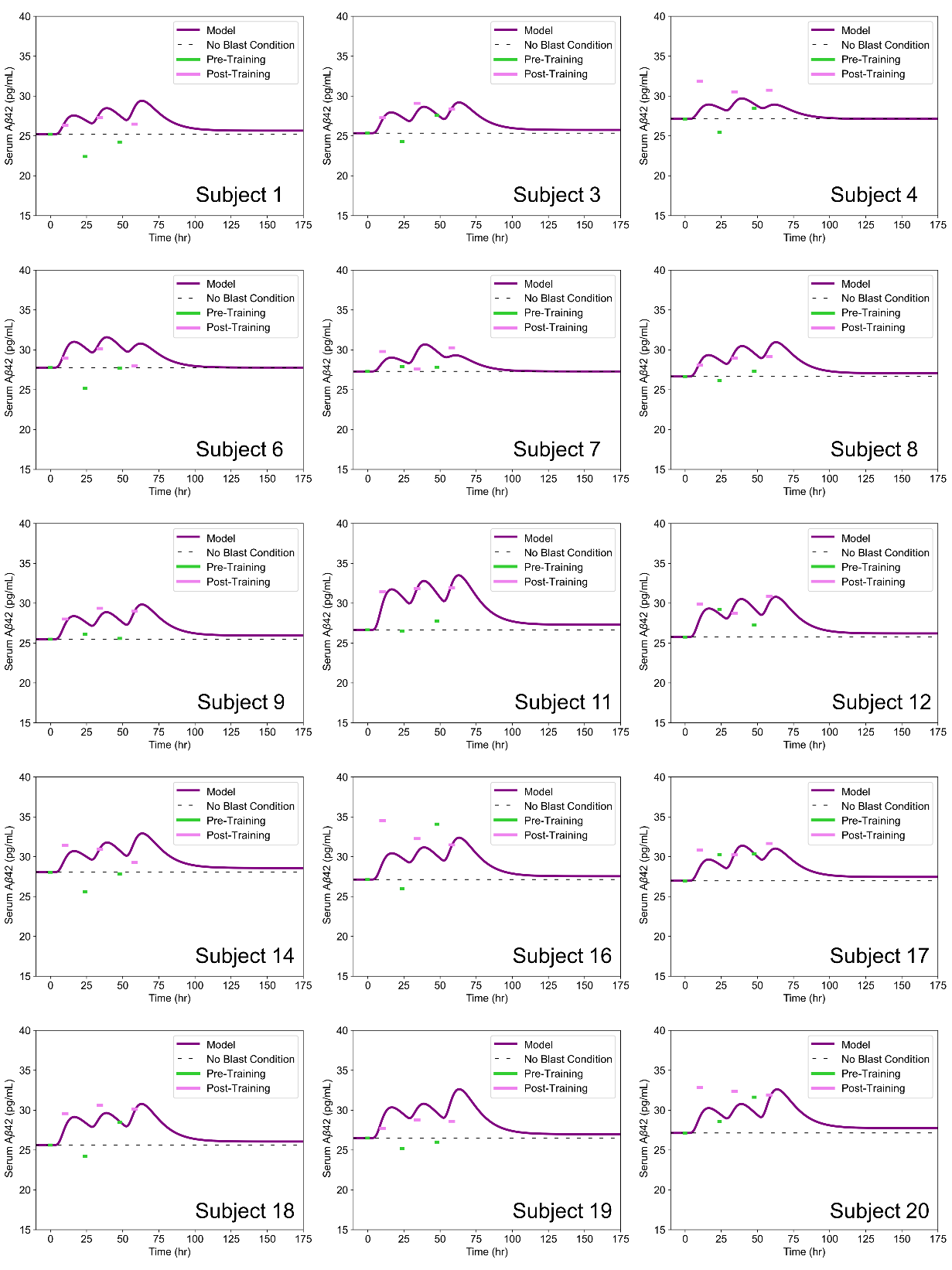


**Supplementary Figure 1.** Blast-Dose BxK model serum Aβ42 concentrations compared to experimental serum data for all subject IDs reported in Thangavelu et al. (24).
