# Supplementary material for: Modeling biomarker kinetics of Aβ levels in serum following blast
Source: Front Neurol. 2025 Apr 4;16:1548589. doi: 10.3389/fneur.2025.1548589 (PMC12006977; doi:10.3389/fneur.2025.1548589)
Supplement: Supplementary file 2 [file Table_1.DOCX]

Supplementary Material

**Supplementary Table 1.** Reaction mechanisms.

| **Brain** | | **Tissue** |
| --- | --- | --- |
| *Brain Vascular* | *ISF* | *Tissue Vascular* |
| $M_{\mathrm{bv}}\underset{\to}{Q_{\mathrm{bv}}{-L}_{\mathrm{bv}}}M_{bl}$ | $M_{\mathrm{bv}}\underset{\to}{Q_{bv,i}(1-\sigma_{bv,i})}M_{i}$ | $M_{bl}\underset{\to}{Q_{t}}M_{\mathrm{tv}}$ |
| $M_{bl}\underset{\to}{Q_{\mathrm{bv}}}M_{\mathrm{bv}}$ | $M_{\mathrm{bb}}\underset{\to}{F_{bb,i}}M_{i}$ | $M_{\mathrm{tv}}\underset{\to}{Q_{t}{-L}_{t}}M_{bl}$ |
| $M_{\mathrm{bv}}\underset{\to}{Q_{bv,c}(1-\sigma_{bv,c})}M_{c}$ | $M_{i}\underset{\to}{F_{i,bb}}M_{\mathrm{bb}}$ | $M_{\mathrm{tv}}\underset{\to}{L_{t}(1-\sigma_{tv,t})}M_{t}$ |
| $M_{\mathrm{bv}}\underset{\to}{Q_{bv,i}(1-\sigma_{bv,i})}M_{i}$ | $M_{i}\underset{\to}{Q_{i,pv}(1-\sigma_{i,pv})}M_{\mathrm{pv}}$ | $M_{\mathrm{tv}}\underset{\to}{F_{tv,tvb}}M_{\mathrm{tvb}}$ |
| $M_{\mathrm{bv}}\underset{\to}{F_{bv,cb}}M_{\mathrm{cb}}$ | $M_{i}\underset{\to}{Q_{i,L}(1-\sigma_{i,L})}M_{L}$ | $M_{\mathrm{tvb}}\underset{\to}{F_{tvb,tv}}M_{\mathrm{tv}}$ |
| $M_{\mathrm{bv}}\underset{\to}{F_{bv,bb}}M_{\mathrm{bb}}$ | $M_{i}\underset{\to}{Q_{i,c}}M_{c}$ | $M_{\mathrm{tv}}\underset{\to}{\mathrm{Cl}_{\mathrm{tv}}}0$ |
| $M_{\mathrm{cb}}\underset{\to}{F_{cb,bv}}M_{\mathrm{bv}}$ | $M_{c}\underset{\to}{Q_{c,i}}M_{i}$ | *Tissue Vascular Barrier* |
| $M_{\mathrm{bb}}\underset{\to}{F_{bb,bv}}M_{\mathrm{bv}}$ | *CSF* | $M_{\mathrm{tv}}\underset{\to}{F_{tv,tvb}}M_{\mathrm{tvb}}$ |
| $M_{\mathrm{bv}}\underset{\to}{\mathrm{Cl}_{\mathrm{bv}}}0$ | $M_{\mathrm{bv}}\underset{\to}{Q_{bv,c}(1-\sigma_{bv,c})}M_{c}$ | $M_{\mathrm{tvb}}\underset{\to}{F_{tvb,t}}M_{t}$ |
| *BBB* | $M_{\mathrm{cb}}\underset{\to}{F_{cb,c}}M_{c}$ | $M_{t}\underset{\to}{F_{t,tvb}}M_{\mathrm{tvb}}$ |
| $M_{\mathrm{bv}}\underset{\to}{F_{bv,bb}}M_{\mathrm{bb}}$ | $M_{c}\underset{\to}{F_{c,cb}}M_{\mathrm{cb}}$ | $M_{\mathrm{tvb}}\underset{\to}{F_{tvb,tv}}M_{\mathrm{tv}}$ |
| $M_{\mathrm{bb}}\underset{\to}{F_{bb,i}}M_{i}$ | $M_{c}\underset{\to}{Q_{c,L}(1-\sigma_{c,L})}M_{L}$ | $M_{\mathrm{tvb}}\underset{\to}{\mathrm{Cl}_{\mathrm{tvb}}}0$ |
| $M_{\mathrm{bb}}\underset{\to}{F_{bb,bv}}M_{\mathrm{bv}}$ | $M_{i}\underset{\to}{Q_{i,c}}M_{c}$ | *Tissue* |
| $M_{i}\underset{\to}{F_{i,bb}}M_{\mathrm{bb}}$ | $M_{c}\underset{\to}{Q_{c,i}}M_{i}$ | $M_{\mathrm{tv}}\underset{\to}{L_{t}(1-\sigma_{tv,t})}M_{t}$ |
| $M_{\mathrm{bb}}\underset{\to}{\mathrm{Cl}_{\mathrm{bb}}}0$ | $M_{c}\underset{\to}{Q_{c,pv}(1-\sigma_{c,pv})}M_{\mathrm{pv}}$ | $M_{\mathrm{tvb}}\underset{\to}{F_{tvb,t}}M_{t}$ |
| *PVS* | *BCSFB* | $M_{t}\underset{\to}{F_{t,tvb}}M_{\mathrm{tvb}}$ |
| $M_{i}\underset{\to}{Q_{i,pv}(1-\sigma_{i,pv})}M_{\mathrm{pv}}$ | $M_{\mathrm{bv}}\underset{\to}{F_{bv,cb}}M_{\mathrm{cb}}$ | $M_{t}\underset{\to}{L_{t}(1-\sigma_{t,L})}M_{L}$ |
| $M_{c}\underset{\to}{Q_{c,pv}(1-\sigma_{c,pv})}M_{\mathrm{pv}}$ | $M_{\mathrm{cb}}\underset{\to}{F_{cb,c}}M_{c}$ | $M_{t}\underset{\to}{\mathrm{Cl}_{t}}0$ |
| $M_{\mathrm{pv}}\underset{\to}{Q_{pv,L}(1-\sigma_{pv,L})}M_{L}$ | $M_{\mathrm{cb}}\underset{\to}{F_{cb,bv}}M_{\mathrm{bv}}$ |  |
|  | $M_{c}\underset{\to}{F_{c,cb}}M_{\mathrm{cb}}$ | **Blood** |
|  | $M_{\mathrm{cb}}\underset{\to}{\mathrm{Cl}_{\mathrm{cb}}}0$ | $M_{bl}\underset{\to}{Q_{\mathrm{bv}}}M_{\mathrm{bv}}$ |
| **Lymph** | | $M_{\mathrm{bv}}\underset{\to}{Q_{\mathrm{bv}}{-L}_{\mathrm{bv}}}M_{bl}$ |
| $M_{L}\underset{\to}{L_{\mathrm{bv}}{+L}_{t}}M_{bl}$ | | $M_{bl}\underset{\to}{Q_{t}}M_{\mathrm{tv}}$ |
| $M_{t}\underset{\to}{L_{t}(1-\sigma_{t,L})}M_{L}$ | | $M_{\mathrm{tv}}\underset{\to}{Q_{t}{-L}_{t}}M_{bl}$ |
| $M_{\mathrm{pv}}\underset{\to}{Q_{pv,L}(1-\sigma_{pv,L})}M_{L}$ | | $M_{L}\underset{\to}{L_{\mathrm{bv}}{+L}_{t}}M_{bl}$ |
| $M_{i}\underset{\to}{Q_{i,L}(1-\sigma_{i,L})}M_{L}$ | | $M_{bl}\underset{\to}{\mathrm{Cl}_{bl}}0$ |
| $M_{c}\underset{\to}{Q_{c,L}(1-\sigma_{c,L})}M_{L}$ | |  |
| $M_{L}\underset{\to}{\mathrm{Cl}_{L}}0$ | |  |
