# Supplementary material for: Modeling biomarker kinetics of Aβ levels in serum following blast
Source: Front Neurol. 2025 Apr 4;16:1548589. doi: 10.3389/fneur.2025.1548589 (PMC12006977; doi:10.3389/fneur.2025.1548589)
Supplement: Supplementary file 3 [file Table_2.DOCX]

Supplementary Material

**Supplementary Table 2.** Blast-dose BxK model parameter assumptions.

| *Reflection Coefficients* | | *Clearance* [1/s] | |
| --- | --- | --- | --- |
| $\sigma_{pv,L}$ | 0.65 | $\mathrm{Cl}_{\mathrm{bv}}$ | 3.33e-5 |
| $\sigma_{i,L}$ | 0.65 | $\mathrm{Cl}_{\mathrm{tv}}$ | 1.90e-4 |
| $\sigma_{c,L}$ | 0.65 | $\mathrm{Cl}_{\mathrm{bb}}$ | 7.39e-3 |
| $\sigma_{t,L}$ | 0.2 | $\mathrm{Cl}_{\mathrm{tvb}}$ | 7.39e-3 |
| $\sigma_{i,pv}$ | 0.2 | $\mathrm{Cl}_{t}$ | 1.9e-4 |
| $\sigma_{tv,t}$ | 0.9233 | $\mathrm{Cl}_{\mathrm{cb}}$ | 7.39e-3 |
| $\sigma_{bv,i}$ | 0.975 | $\mathrm{Cl}_{bl}$ | 1.9e-4 |
| $\sigma_{bv,c}$ | 0.975 | $\mathrm{Cl}_{L}$ | 1.9e-4 |
| $\sigma_{c,pv}$ | 0.2 |  | |
| *Flow Rates* [L/h] | | *Flux* [1/s] | |
| $L_{\mathrm{bv}}$ | 0.0345 | $F_{bv,cb}= F_{bv,bb}$ | 1.72e-9 |
| $L_{t}$ | 0.321 | $F_{cb,bv}= F_{bb,bv}$ | 4.5e-5 |
| $Q_{bl}$ | 182.07 | $F_{c,cb}= F_{i,bb}$ | 1.48e-7 |
| $Q_{\mathrm{bv}}= (1-0.882)Q_{bl}$ | 21.5 | $F_{cb,c}= F_{bb,i}$ | 1.48e-8 |
| $Q_{t}= (0.882)Q_{bl}$ | 160.5 | $F_{tv,tvb}= F_{t,tvb}$ | 3.0e-6 |
| $Q_{i,c}$ | 0.0105 | $F_{tvb,tv}= F_{tvb,t}$ | 2.0e-5 |
| $Q_{c,i}$ | 0.0105 | *Volumes* [L] | |
| $Q_{bv,c}$ | 0.024 | $V_{bl}$ | 2.9155 |
| $Q_{bv,i}$ | 0.024 | $V_{\mathrm{tv}}$ | 1.68 |
| $Q_{i,L}= Q_{bv,i}- Q_{i,pv}$ | 0.0219 | $V_{\mathrm{tvb}}$ | 0.335 |
| $Q_{pv,L}=Q_{c,pv}+ Q_{i,pv}$ | 0.00234 | $V_{t}$ | 11.1 |
| $Q_{c,L}= Q_{bv,c}- Q_{c,pv}$ | 0.02376 | $V_{\mathrm{bv}}$ | 0.0319 |
| $Q_{i,pv}=(8.75/100)Q_{bv,i}$ | 0.0021 | $V_{\mathrm{bb}}$ | 0.00659 |
| $Q_{c,pv}=(Q_{bv,c}/100)$ | 0.00024 | $V_{\mathrm{cb}}$ | 0.000659 |
| *Physiological Constants: Male* | | $V_{i}$ | 0.261 |
| Body Weight [kg] | 70 | $V_{c}$ | 0.143 |
| Cardiac Output [L/min] | 5.1 | $V_{L}$ | 0.274 |
| Hematocrit | 0.405 | $V_{\mathrm{pv}}$ | 0.00235 |
| Blood Volume [L] | 4.9 |  | |
| *Initial Conditions* [nMol/L] | | | |
| ${A\beta42}_{i}$ | 0.0238 | ${C99}_{i}$ | 0.4221 |
| ${A\beta40}_{i}$ | 0.73444 | ${C99}_{t}$ | 0.4221 |
